# Prostate organoids: emerging experimental tools for translational research

**DOI:** 10.1172/JCI169616

**Published:** 2023-05-15

**Authors:** Michael Beshiri, Supreet Agarwal, Juan Juan Yin, Kathleen Kelly

**Affiliations:** Laboratory of Genitourinary Cancer Pathogenesis, Center for Cancer Research, National Cancer Institute, Bethesda, Maryland, USA.

## Abstract

Organoid technology has provided new translational research opportunities in oncology, in part by enabling the development of patient-representative living biobanks. Prostate cancer research historically has been constrained to a small number of in vitro models, limiting the ability to translate experimental conclusions for contemporary, heterogeneous patient populations. The facility of organoid culture methods to maintain luminal prostate epithelia, the common lineage of prostate cancers, has greatly expanded the phenotypic and genotypic diversity of available tractable models, including luminal stem/progenitor cells and progressive patient-derived cancers. Biobanks of patient prostate cancer organoids enable increased accuracy in predicting therapeutic efficacy and informative clinical trial designs. Here, we discuss how prostate organoid technology is currently being used, the promising areas of future therapeutic applications, and the current obstacles to be overcome.

## Introduction

Prostate cancer is the second most common form of cancer in men in the United States, estimated to affect more than 288,000 men in 2022, with 34,700 predicted to die from the disease (1). The vast majority of prostate cancers are adenocarcinomas of a luminal phenotype that express the transcription factor androgen receptor (AR) and are highly dependent on AR signaling. Prostate luminal epithelium, which can be divided further into subtypes, composes the inner layer of prostate tubules and acinar glands, while basal cells line the supportive base of these structures. Localized prostate cancer is highly treatable with surgery or radiation, but approximately 20% of men experience lethal recurrence (2). Recurrent disease is treated with AR signaling inhibition, resulting almost always in progression to metastatic, castration-resistant prostate cancer (CRPC) (3). CRPC usually remains an AR-dependent adenocarcinoma, but about 20% of the time, plasticity to other differentiated cell types is observed, including the expression of a neuroendocrine-lineage phenotype (4, 5). Insight into the genetic determinants of prostate cancer has come from multi-institutional coordinated efforts to sequence large sets of patient tissue samples, and CRPC has proven to be highly genetically diverse (6–8).

Investigations encompassing the cellular and molecular characteristics of prostate cancer are important in deciphering mechanisms related to tumorigenesis, prostate cancer progression, and treatment vulnerability/resistance, and in establishing new therapeutic approaches. Such investigations require representative and experimentally tractable preclinical models. Unfortunately, the complement of in vitro models of prostate cancer until recently has been limited to a small number of cell line models, reflecting only a fraction of the phenotypic and genotypic diversity of the disease (9, 10). Furthermore, the normal counterpart of prostate cancer, luminal prostate epithelium, historically has been refractory to growth in culture, while prostate basal epithelium is more readily propagated.

## History and current status of prostate organoid culturing

Recent advances in ex vivo techniques have increased the number and improved the representational quality of preclinical models across cancer research (11). In an effort to overcome the inherent resistance of normal and transformed prostate luminal epithelium to traditional 2D culturing, the field has incorporated 3D organoid culture methods (12–15). The term “organoid” has been used for decades in association with 3D culture techniques without a strict definition (16). It has encompassed embedding within a 3D extracellular matrix (ECM) tissue fragment explants, induced and embryonic pluripotent stem cells, and adult tissue stem cells (16). Organoids generally are self-organizing structures or mini-organs clonally generated from a cell capable of producing differentiated phenotypes.

Embedding cells within an ECM promotes spatial organization and mechanosensory signaling more similar to normal tissue structures than monolayers grown on plastic. Because competition for 2D adhesive surfaces is not a factor, there is less selection for the most rapidly growing cells, better preserving the cellular heterogeneity associated with differentiating and/or cancer cell populations. Consistent with this, analyses of CRPC organoid clonal heterogeneity relative to serial passages have revealed model-specific stability, with a relatively modest loss of clonal variability, in the limited number of models that have been analyzed (17). As a continuously growing, renewable source of cells, organoids present tractable platforms for various cell biological, biochemical, and genetic manipulations, enabling mechanistic studies.

The initial advancements to prostate 3D culture methods were made using mouse-derived spheroid culture assays. These methods cultured whole or fractionated mouse prostate epithelium in a commercially available, defined, serum-free medium and in a 3D ECM, demonstrating spheroids with self-renewing and self-organizing differentiation activities (18–20). These culture conditions favored mouse basal cells and generally were not sufficient for culturing human prostate cancer (21). Such approaches capitalized on the interaction between ITGA6-expressing prostatic stem cells and the laminin component of the ECM in Matrigel. The use of Matrigel in the culture assays was key to this early success and remains so in the more recent methods (19, 21, 22). This early approach allowed continued propagation of TP63^+^ basal cells but lacked neuroendocrine and luminal cell types. Addition of androgen such as dihydrotestosterone (DHT) to the medium drove some limited luminal differentiation to intermediate phenotypes with AR expression, but these spheroids still lacked the secretory phenotype indicative of functional prostate luminal epithelium (22).

In 2009 Sato et al. defined organoid medium conditions incorporating stem cell niche factors, including the Wnt pathway agonist R-spondin-1, EGF, and the BMP antagonist Noggin (23). This method supported the long-term self-renewal and differentiation capacity of mouse intestinal crypt stem cells in a 3D ECM. Modification to the base medium with the addition of nicotinamide, prostaglandin E_2_, and inhibition of TGF-β and MAPK14 signaling allowed for culture of human intestinal organoids to be established from intact intestinal crypts as well as isolated stem cells, derived from either normal or tumor tissues (24). Variations on these stem cell–promoting culture conditions have proven useful to establish the growth of various epithelial cancers, often after optimization with tissue-specific factors, e.g., the addition of estrogen or testosterone in the case of certain subtypes of breast or prostate cancers, respectively (14, 25). Importantly, organoid cultures establish autonomous growth of epithelial cells in the absence of additional cell types.

This approach has been adapted to the normal prostate epithelium and prostate cancer with some success (14). Initial work from the Clevers and Sawyers laboratories demonstrated the continuous growth and differentiation of prostate luminal epithelial mouse stem cells and the role of R-spondin, Noggin, and testosterone as facilitating but not required for growth (14). Prostate-specific modification of the intestinal medium conditions and other variations on 3D culture procedures (15) have allowed the robust culture of mouse-derived prostate organoids, including normal luminal cells (26).

Yu Chen and colleagues first described the utility of organoid culturing for establishing models from patient-derived metastatic CRPC tissue (14). Although variable success rates in establishing long-term culture from patient tumor needle biopsies have been reported, an average rate of 10% or less is significantly lower than the rates for many other epithelial tumors, such as pancreatic, colorectal, or breast, reflecting CRPC tumor heterogeneity as well as incompletely optimized culture conditions (11). Fujii et al. showed for colorectal cancer (CRC) that different genetic backgrounds within the same cancer type affect the specific culture requirements of the patient-derived organoids (27). In these models, dependence on specific factors to maintain a niche-like microenvironment was reduced when comparing normal tissue with tumor and further reduced as tumors progressed from early to late clinical stage. Independence from Wnt and R-spondin supplementation reliably tracked with mutations in the Wnt pathway, while the loss of a requirement for TGF-β inhibition was linked to mutations in the matching pathway at a rate approaching 40%. Additional niche requirements like EGF supplementation and MAPK14 inhibition could often, but not always, be eliminated based on a known underlying genetic mechanism. Similarly in the prostate field, there exists no single defined medium formulation that is sufficient or necessary to support the growth of all prostate cancer organoid models, but unlike in CRC, no correlations have been determined thus far between genotypes and different medium requirements (17, 28). Despite the persistent challenges, a diverse and growing set of new models have been developed as prostate cancer organoids from metastatic tissue biopsies. These new models represent well-known CRPC phenotypes, including AR-driven (AR^+^) adenocarcinoma and AR-independent neuroendocrine-positive (NE^+^) or double-negative (AR^–^NE^–^) lineages (14, 17, 29, 30). In addition, a previously unrepresented subtype, an AR^+^NE^+^ amphicrine model, which demonstrates continuous multilineage terminal differentiation from progenitor cells, has been described (31). Also, an organoid model derived from and reflecting a treatment-naive (castration-sensitive) state has been established (32). In addition, modified culture conditions have improved the adaptation of patient-derived xenograft (PDX) tissue to organoid culture, expanding further the diversity of patient-derived models that can be manipulated in culture (17).

Despite the enlarging compendium of CRPC models enabled by organoid culturing techniques, establishing primary prostate cancer models has remained an intractable problem. Careful analyses for the presence of driver mutations in the initiating primary prostate cancer samples compared with the resulting organoid cultures have demonstrated that primary luminal cancers coexist initially with normal basal cells. Within a relatively few passages, however, basal cells become the prominent cellular component of such cultures (28). The vigorous growth of basal epithelial cells in organoid cultures can be misleading and demonstrates the need for genetic verification of prostate cancer cellular growth.

## Investigations using mouse prostate organoids

The ability to culture a range of normal and transformed prostate epithelial cells from primary mouse tissues has enabled important advancements in our understanding of prostate cancer biology. The identity and properties of regenerative cells that contribute to pathological conditions, including as one proposed cell of origin for prostate cancer, have been of intense interest for decades. Organoid culture conditions support stem cell self-renewal and differentiation, providing assays to assess the number and potentiality of stem/progenitor cells during development, homeostasis, castration/injury, and cancer development (33–35).

A consistent finding among investigations into the characterization of mouse prostate stem cells, often using complementary approaches, has been the distinction relative to the urethra of proximal (ductal) versus distal (acinar) populations as enriched for castration-resistant luminal stem/progenitor cells versus predominantly castration-sensitive, differentiated luminal cells, respectively (33). A number of recent single-cell RNA sequencing analyses have refined the characteristics of mouse prostate epithelial cells relative to individual markers and, in some studies, relative to anatomical locations (33, 36–38). Among various naming conventions, the proximal *Trop2^+^*, SCA^hi^ luminal population has been called Lum2 or LumP, and the distal population Lum1 or designations relative to the specific distal lobe (see ref. 39 for a comparative table). In addition, a distinct urethra-adjacent population expressing both basal and luminal markers (PrU) has been described and may represent the most primitive multipotent progenitors (37, 40). Basal, Lum2/LumP, and PrU populations demonstrate the highest frequencies of organoid formation and bipotent differentiation potential as well as the most robust grafting potential in the presence of fetal urogenital mesenchyme (37). By comparison, distal luminal cells form fewer organoids and require more cells for successful grafting. A precise analogy between mouse and human prostate epithelial populations currently is not entirely clear, although ductal and acinar population symmetry exists (41). Also, the organoid-forming properties of human cell populations defined by single-cell transcriptomes have not been described. Importantly, analyses of organoid-forming frequencies compared with in vivo methods of lineage tracing in the mouse have indicated that the progenitor potential revealed by dissociated cells in organoid culture is restricted by context in situ (39). For example, proximal cells are spatially restricted and do not contribute to distal acinar regeneration (42).

Organoid cultures have great potential in modeling the genetic basis of prostate cancer biology. Specifically, it has been possible to culture genetically engineered mouse models spanning the spectrum of prostate cancer phenotypes, including indolent/low-grade prostatic intraepithelial neoplasia, well-differentiated and poorly differentiated adenocarcinoma, and neuroendocrine cancer (14, 15, 26, 43, 44). Interestingly, organoids derived from distinct tumor histologies often recapitulate aspects of the in vivo morphology, such as multilayering or invasive edges, exemplifying how organoids identify tumor-autonomous properties (45).

Various molecular and cellular properties associated with genetic alterations in prostate cancer can be modeled by direct alteration of appropriate cells whose growth is enabled by organoid culturing. The ability to manipulate luminal prostate epithelium in culture with lentiviral infection has allowed for early as well as transient states to be modeled following Cre- or CRISPR-mediated genomic rearrangements or ectopic expression of mutant proteins. For example, mutations in *SPOP*, which occur early in the development of prostate cancer, are most appropriately modeled starting with normal luminal stem cells, which provide an appropriate context, as compared with prostate cancer cell lines, which contain several preexisting mutations (43). In summary, the availability of a tractable culture system for the in vitro growth of a variety of progenitor and differentiated prostate epithelial cells has facilitated the identification and molecular characterization of normal and transformed subpopulations as well as transient changes that occur in these subpopulations (46), deepening our understanding of the mechanisms of transformation underpinning prostate cancer.

## Investigations using human prostate organoids

Investigations into patient-derived prostate cancer historically have been limited to a small number of cell lines that fail to represent the heterogeneous phenotypic and genotypic diversity observed in patient populations. As described earlier, the addition of new models from patient CRPC samples has been measurable but slow. Nonetheless, several new models derived from the direct organoid culturing of patient biopsies have been established, and transcriptomic and chromatin accessibility analysis of some of these has identified previously unknown Wnt-dependent and AP-1–driven subtypes of adenocarcinoma (30) and has added new transitional small-cell neuroendocrine (tSCNPC) (29) and amphicrine models (17).

Organoid cultures from CRPC biopsy samples demonstrate highly variable growth rates, presumably reflecting the heterogeneity of phenotypes observed in patients. Compared with the efficient establishment and the rapid growth of some cancer types, such as gastrointestinal cancers, CRPC organoids require several weeks and sometimes months to expand sufficiently to be characterized and used for functional assays (17, 28). Therefore, both low efficiency of establishment and slow growth rates make personalized medicine approaches for individual patients relatively impractical, limiting the testing of “avatar samples” directly for therapeutic responsiveness. Alternatively, the construction and analysis of large, representative CRPC organoid cohorts have the potential to identify classes of responders and nonresponders, associated response mechanisms, and potential predictive biomarkers.

Importantly, PDX cohorts of CRPC and in some cases primary prostate cancer have been established at various centers over the past two decades (47). Cultures of CRPC PDX-derived organoids provide additional well-characterized, widely available models, which are genetically and phenotypically heterogeneous and representative. PDX-derived organoid approaches have advantages and disadvantages. One notable technical advantage is the ability to harvest large numbers of tumor cells at one time, which is often an expensive challenge with slow-growing organoid cultures. Disadvantages include prior selection in the mouse, although PDX tumors do maintain a substantial amount of heterogeneity, which is captured in organoid cultures (17, 48). In addition, PDX models introduce mouse cell contamination, which is best addressed with species-specific purification methods prior to organoid culturing. Although as many as half of PDX models cannot be maintained long-term (more than five passages) in organoid culture, initial growth for 10 to 14 days is sufficient for drug screening purposes (17). Combined with patient-derived organoids (PDOs) from biopsies, PDX-derived organoids substantially expand the diversity of tractable CRPC models.

**Figure 1:**
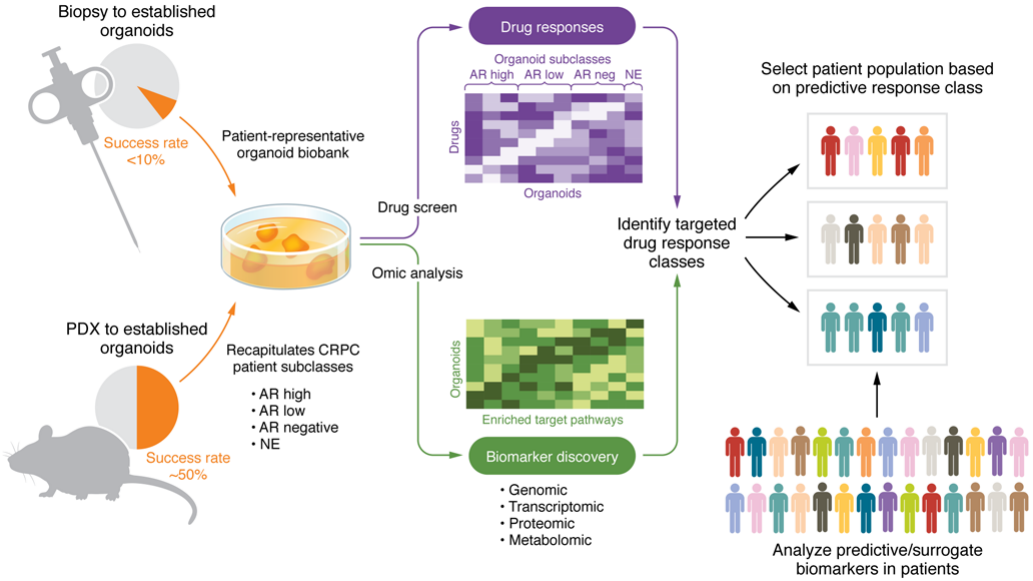
From CRPC organoid to clinical trial. Patient biopsy and PDX tissues are used to establish prostate cancer organoids that recapitulate subclasses of patients with CRPC. Patient-representative organoid banks can be used for high-throughput drug screening and comprehensive biomarker detection. Following marker determinations, integrated data can be used to elucidate the most promising patient populations to consider. neg, negative.

Representative cohorts that address potential frequencies and specificities of therapeutic response relative to various genomic and phenotypic biomarkers are particularly important for the effective translation of drug screening to patient populations, as shown by the predictive utility of tumor organoids for patient responses in other cancer types (45, 49–51) (Figure 1). Organoids are amenable to testing of small molecules, biological therapeutics such as antibody-based treatments, and cell-based therapies. One of the first examples of such a comprehensive analysis has been performed for an antibody-directed cytotoxic drug specific for B7H3/CD276, a tumor antigen that is expressed on a number of solid tumor types, and containing a pyrrolobenzodiazepine cytotoxic moiety (52). Responders were identified across various histological phenotypes of 27 CRPC organoid models, as was a class of B7H3^+^ adenocarcinoma nonresponders. RB1 loss, SLFN11 expression, and IFN response gene signatures were observed to be biomarkers of responsiveness. On the other hand, levels of B7H3 expression were necessary, but not sufficient, and did not quantitatively correlate with responsiveness. Such a study illustrates the heterogeneity of CRPC, underscoring the importance of extensive analyses, and demonstrates the ability to identify mechanistic subclasses outside of the typical histological or specific target designation.

It is anticipated that knowledge concerning the specificity of CRPC responses to therapeutics will improve the design and efficacious outcomes of clinical trials. The information obtained by combining quantitative drug responses from a variety of patient-representative models with biomarker correlates, including genotypic, transcriptomic, and protein markers as well as pathological features, not only identifies potentially efficacious drugs but suggests appropriate patient populations as well as related biomarkers to include in the trial design. Prospective clinical trials are required to determine the drug classes and CRPC subtypes for which organoids are most predictive of patient responses.

## Future considerations

The availability of a wide array of naturally occurring genetic mutations in CRPC organoids has the potential to provide new platforms that can be used for testing of mechanistic hypotheses and discovery via molecular analyses such as gene-based screens or in response to environmental manipulation. Another area of great potential is the development of culture conditions that support prostate epithelial cells in combination with cells in the normal or tumor microenvironment (TME), including stromal and immune populations. A reductionist approach allows modeling of the effects of specific components of the TME on the growth of organoids derived from prostate normal epithelium or tumors. For example, investigations have shown a contribution of the stromal- and immune-derived factor NRG1 to survival of prostate luminal progenitors in response to androgen signaling inhibitors and the development of CRPC (51, 53).

In other cancer types, organoid models are proving highly useful in both the evaluation of and potential discovery related to immuno-oncology therapeutics. Tumor organoids have been used in combination with matching patient peripheral blood lymphocytes to measure the existing cytotoxic activity in response to immuno-oncology drugs as well as to amplify and identify relevant T cell antigen receptors (54–56). Similarly to challenges encountered using patient tumor avatars for drug screens, the low frequency of prostate cancer organoid establishment is a major impediment. However, as highly enriched tumor cells reflecting the heterogeneity and cellular phenotypes of naturally occurring tumors, organoids provide a useful source of relevant tumor antigens and can be exploited as targets for engineered chimeric antigen receptor (CAR) T cells or to identify tumor-specific neoantigens (57).

Finally, identifying the restrictive factors that lead to inefficient organoid establishment from prostate cancers and developing approaches to overcome this current challenge remain important goals. Prostate stroma is thought to be a critical component in mediating responsiveness to androgen deprivation, suggesting an androgen-responsive growth-promoting cell in the prostate microenvironment (58). Likewise, metastatic prostate cancer almost always homes to the bone, suggesting the existence of a growth-supportive cell in the bone marrow (59, 60). Identifying and utilizing growth-supportive cells in organoid cocultures is one potential approach, as well as consideration of further medium and ECM optimizations.

## Conclusion

Despite the ample space left for development, organoid technology as it exists today has great potential to be highly impactful in translational prostate cancer research. Considering the paucity of in vitro models of prostate cancer, the representational quality of patient-derived models allows for the study of relevant genotypes and phenotypes observed in patients that are otherwise not readily accessible. The impact of patient-derived organoid models and the acceleration of their use will best be realized through the establishment of biobanks available to the community. An accurate representation of contemporary patient populations combined with their experimental tractability makes organoids particularly useful for mechanistic studies, biomarker identification, and evaluation of drug responses.

## Version 1 05/15/2023

Electronic publication
